# Supplementary material for: Optical trapping of otoliths drives vestibular behaviours in larval zebrafish
Source: Nat Commun. 2017 Sep 20;8:630. doi: 10.1038/s41467-017-00713-2 (PMC5606998; doi:10.1038/s41467-017-00713-2)
Supplement: Supplementary file 1 — Supplementary Information [file 41467_2017_713_MOESM1_ESM.pdf]

Description of Supplementary Files

File Name: Supplementary Information
Description: Supplementary Figures, Supplementary Methods and Supplementary References

File Name: Supplementary Movie 1
Description: **Manipulation of a freely moving otolith, dissected from a larva.** A 250 mW laser power OT is sufficient to drag the otolith around a dish. A 100 µm grid provides scale.

File Name: Supplementary Movie 2
Description: **Tail responses to OTs of varying power.** An optical trap is applied on the lateral edge of the left otolith at four different powers. This movie shows Fish 2 (Supplementary Figure 4). **Part 1. Tail response to an OT at 600 mW.** At the onset of the OT, the larva produces a strong forward swim and tail deflection to the right. The tail slowly returns to its initial position after the offset of the trap. **Part 2. Tail response to an OT at 400 mW.** At onset, the larva produces a subtle forward swim and tail deflection to the right. The tail slowly returns to its initial position after the offset of the trap. **Part 3. Tail response to an OT at 200 mW.** At onset, the tail deflects weakly to the right, and slowly returns to baseline after the OT ends. **Part 4. Tail response to an OT at 100 mW.** The larva produces a weak tail movement at the onset of the OT, and this returns to baseline after offset.

File Name: Supplementary Movie 3
Description: **Tail response to 600 mW OT at the centre and lateral side of the right otolith. Part 1. Tail response to OT at LOcent at 600 mW.** No deflection occurs during OT. **Part 2 Tail response to OT at LOlat at 600 mW.** Deflection occurs at the onset of the trap in the contralateral direction.

File Name: Supplementary Movie 4
Description: **Tail responses to different combinations of OTs.** An optical trap is applied to the lateral edge of the larva's right otolith (ROlat), the medial edge of the left otolith (LOmed), or both simultaneously. This movie shows Fish 1 (Supplementary Figure 5). **Part 1. Tail response to ROlat at 600 mW.** Deflection occurs at the onset of the trap in the contralateral direction. **Part 2. Tail response to LOmed at 600 mW.** Deflection occurs at the offset of the trap in the ipsilateral direction. **Part 3. Tail response to ROlat and LOmed simultaneously at 600 mW.** Deflection occurs at the onset of the trap in the contralateral direction and actively returns to the starting position at the offset of the trap.

File Name: Supplementary Movie 5
Description: **Eye responses to OTs at various powers. Part 1. Eye responses to LOlat at 600 mW.** At the onset of the OT, both eyes roll strongly in the same direction. The eyes slowly move toward their baseline positions following OT offset. This movie shows Fish 2 (Supplementary Figure 6). **Part 2. Eye responses to LOlat at 400 mW.** Again, both eyes roll in the same direction, but more moderately. They move gradually toward baseline when the OT is released. **Part 3. Eye responses to LOlat at 200 mW.** A similar effect is seen, but the magnitude of the rolling movement is still weaker.

File Name: Supplementary Movie 6
Description: **Eye responses to OT at the centre of the right otolith at 600 mW.** No response occurs. This movie shows Fish 2 (Supplementary Figure 6).

File Name: Peer Review File
Description:

Supplementary Methods

1. Monte Carlo Method for calculation of beam spreading through tissue

A code using the Monte Carlo method for the calculation of beam spreading through brain tissue in zebrafish has been developed previously by our group[3]. For this study, we used the same code and calculated the spreading of the beam at 200 µm depth with 1064nm wavelength. Otoliths are located about 150 µm under the skin however this depth varies from fish to fish. To estimate the maximum beam distortion at the otolith plane for one fish we calculated the distribution of the beam at 200 µm in depth in brain tissue.

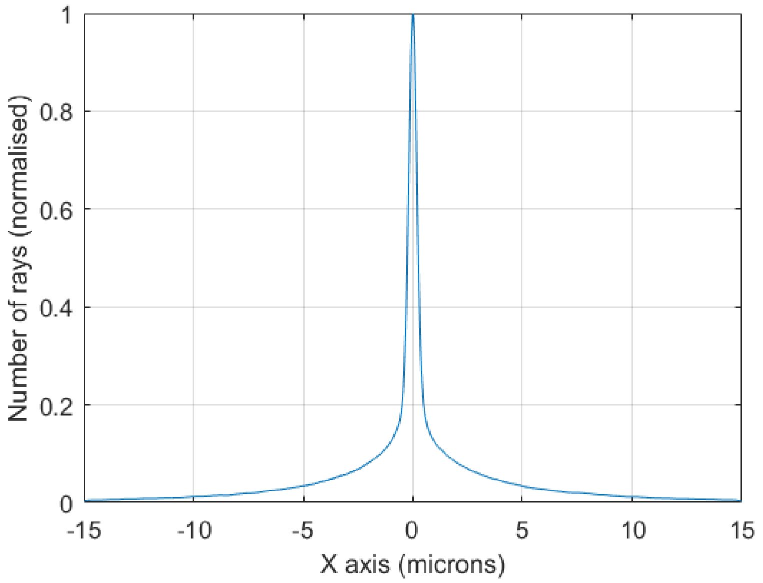

Supplementary Figure 1: Distribution of rays along X axis at 200 µm depth using Monte Carlo method.

The spatial and angular distribution of rays at 200 µm was later used in the ray optics model to calculate the force applied on the otolith *in-vivo* by a scattered focussed beam.

2. Ray optics for theoretical force calculation in otolith

Ray optics calculations are reliable when the wavelength of the radiation incident on the target object is small relative to the object's size, as long as the focus of any beam constructed out of rays is far from the interface between the object and the medium that surrounds it. The ray optics model consists of a beam defined by a Gaussian distribution of rays[4,5]:

$$U_{r,z} = \frac{1}{w_z}\left(\frac{\sqrt{2}r}{w_z}\right)^l e^{-\left(r/w_z\right)^2},$$

where

$$w_z = w_0\sqrt{1 + \left({}^{z}/_{z_R}\right)^2}\ ,$$

and z is the displacement along the beam propagation direction, w_0 is the beam waist at the focus, and r is the transversal position. In this model, rays come from a number of distances, including from the particle boundary, so that the bundle produced better emulates the distribution of light from a paraxial Gaussian beam instead of a ray optical spherical wave coming from far away. To find the forces acting on a spherical particle, only the angle between the external ray and the surface normal of the sphere and the same kind of angle inside the sphere need to be known to calculate the force contribution of each ray.

Another important consideration in the force modelling on otolith is the scattering of light occurring in the brain tissue before reaching the otolith. The ray optics model described above can also incorporate different beam shapes and therefore model the scattering in brain tissue. Using the spatial and angular rays distribution calculated with Monte Carlo method (Supplementary Figure 5) the force calculation with and without scattering (Supplementary Figure 8) shows that, despite the increase in the spot size, the force as a function of photon radiance to reach the otolith is extremely similar to the un-scattered (N.A.=1.0) case. This can be understood through the principle of trapping by the gradient of intensity of the beam. The scatter in the tissue causes a small broadening of the laser spot compared to the particle size and so the rays still experience a large differential in scattering angle due to refraction from one side of the model otolith to the other. A slight reduction of trap force occurs because of the oblique scattering from the statistically distributed trajectories.

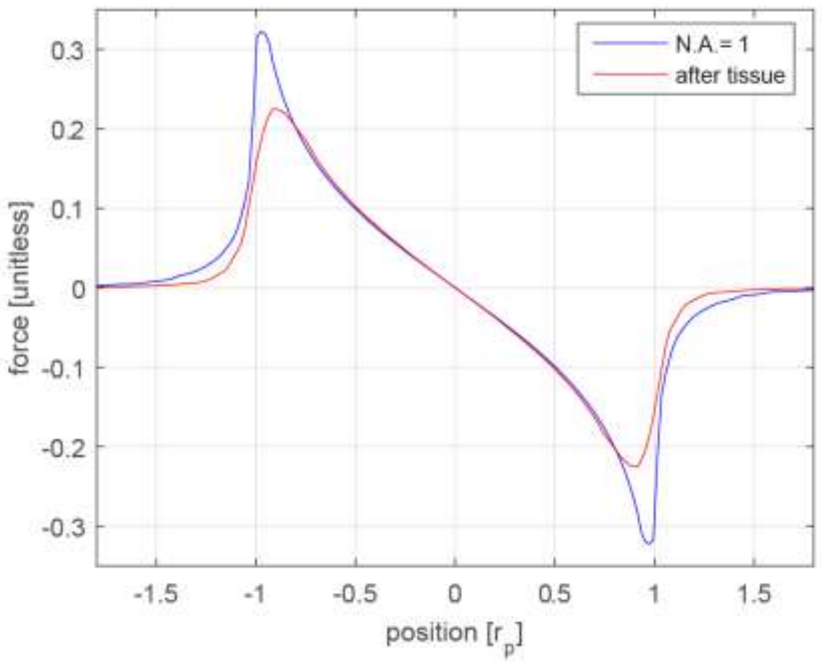

Supplementary Figure 2: Comparison of force per photon for the N.A.=1.0 and tissue scattered N.A.=1.0 case. A slight reduction of the maximum trapping strength is observed due to the scattering mediated defocus of the laser spot.

3. Optical scanning of otolith in water

Scanning of the otoliths in water was performed using the microscope configuration presented in Supplementary Figure 9. Computer control of the stage was achieved with the use of a custom LabVIEW interface to coordinate the movement of the stage and data capture. A raster scan was used to map the force as a function of position.

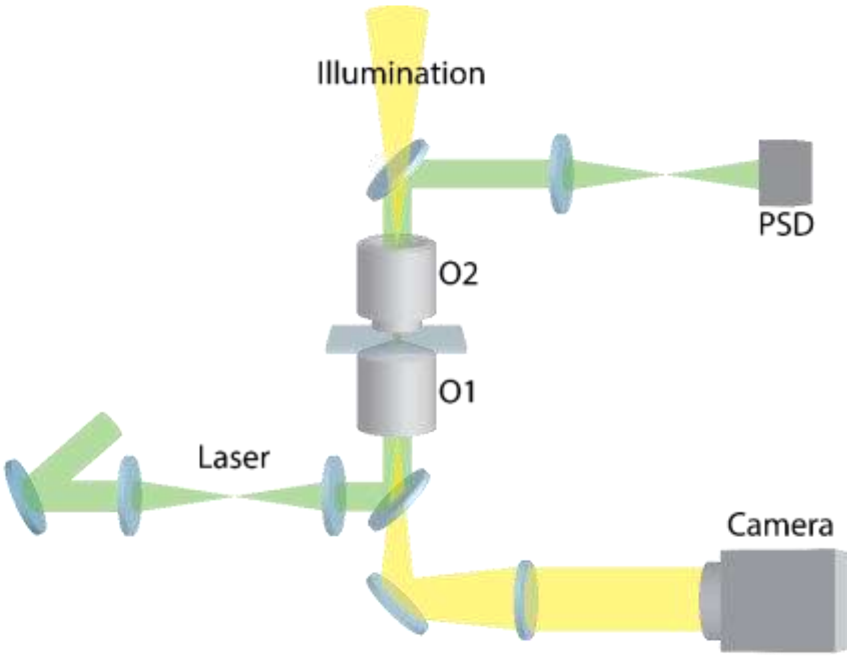

Supplementary Figure 3: Sketch of the setup used for otolith scanning. A static laser (1070nm IPG Photonics YLD-5 fibre laser) was focused by a water immersion objective lens O1 (Olympus UPLSAPO60Xw) to a spot, and a scanning nano stage (PI P-563.3CD) was used to move the otolith. The light deflected by the otolith was collected by a second silicone oil immersion objective lens O2 (Olympus UPLSAPO100XS) and imaged by a transfer lens onto a position sensitive detector PSD (On-track PSM2-10 with OT-301DL amplifier).

The force acting on the particle through scattering is detected by the position sensitive detector by finding the average centre of the scattered laser spot. This is converted to a transverse force (such as x or y) with a scaling factor to convert volts into Newtons. The force acting upon a particle during OT can be related to a dimensionless force with the following expression:

$$F = \frac{nP}{c} Q$$

where F is the force in Newtons, c is the speed of light in vacuum, n is the refractive index, P is the radiant flux of light, i.e., the optical power, and Q is the force contributed per photon momentum.

4. Dual Optical Trapping (OT) setup and imaging

The dual OT system (see Fig 2) is composed of a IR laser (1070nm IPG Photonics YLD-5 fibre laser), a half wave plate that rotates polarisation by 45 degrees, a polarising beam splitter that splits the incoming beam into two beams of same intensity with perpendicular polarisation. Lenses L1 and L2 (100 mm focal length each,) were placed in a 4f configuration (two focal length away from each other) and the two independent beams were reflected off of gimbal mirrors (GM) (Thorlabs GM100). The two beams were recombined with a second polarising beam splitter and a second telescope with lenses L3 (100 mm focal length,) and L4 (200 mm focal length). The beams were then reflected off of a 950 nm cutoff wavelength shortpass dichroic mirror in the imaging column, and projected onto the back focal plane of a 20x 1NA Olympus microscope objective (XLUMPLFLN-W). This created two tightly focussed spots at the imaging plane of the microscope objective. The positions (x,y) of each spot were steered with the GMs, and the third dimension (z) was determined by the distances between the lenses L1 and L2.

The intensities of laser power mentioned in the experiments (0, 100, 200, 400 and 600 mW) were obtained by measuring the intensity of each trap after passing through the optical system described above. The measurements were done using a power meter placed at the focal plane of the 20x 1NA Olympus microscope objective.

The targeting of the traps and imaging of the eyes was done using the same 20x 1NA Olympus objective, a tube lens L5 (180mm focal length, Thorlabs AC508-180-A) and a PCO edge 5.5 camera (Camera 1). To image the whole larva and record its tail movements, a 4x 0.1NA Olympus microscope objective (PLN 4X) was placed below the sample, and a tube lens L6 projected onto a Basler aca1920 camera (Camera 2) recording movements at 160 fps. Note that the 4x and 20x objectives are not collinear.

5. Measuring tail and eye movements

As two focussed spots were targeted independently on the two utricular otoliths, we recorded eye motions at 20Hz using the PCO Edge and tail movements at 160Hz using the Basler aca1920 camera.

Tail bends were imaged along the length of the tail, and clearly visible locations on the tail were tracked horizontally (perpendicular to the tail) using intensity thresholding in Matlab. Finally the ‘smooth’ function was applied on the filtered data using the ‘rloess’ method, a robust local regression using weighted linear least squares and a second degree polynomial model. The angle of deflection was calculated with simple trigonometry by setting the base of the tail as a vertex, the initial position of the tracked marker as one point, and the moving marker as the other point.

Eye motions were tracked in the same dorsal-ventral plane as the utricular otoliths. Visible landmarks on the pigmented retinal epithelium were used to track consistent locations. The displacements of these landmarks were measured by recording centre points of the landmarks, and the value for a given eye in a given trial was the average movement of the landmarks on that eye. The same Matlab code used for the tail was used for the eyes. Roll angles were calculated with simple trigonometry, using 300 µm as the average height of 6dpf embryo eyes, and assuming rotation around the centre of the eye. The laser power and timing of trials was the same during eye tracking as it was for tail tracking, above.

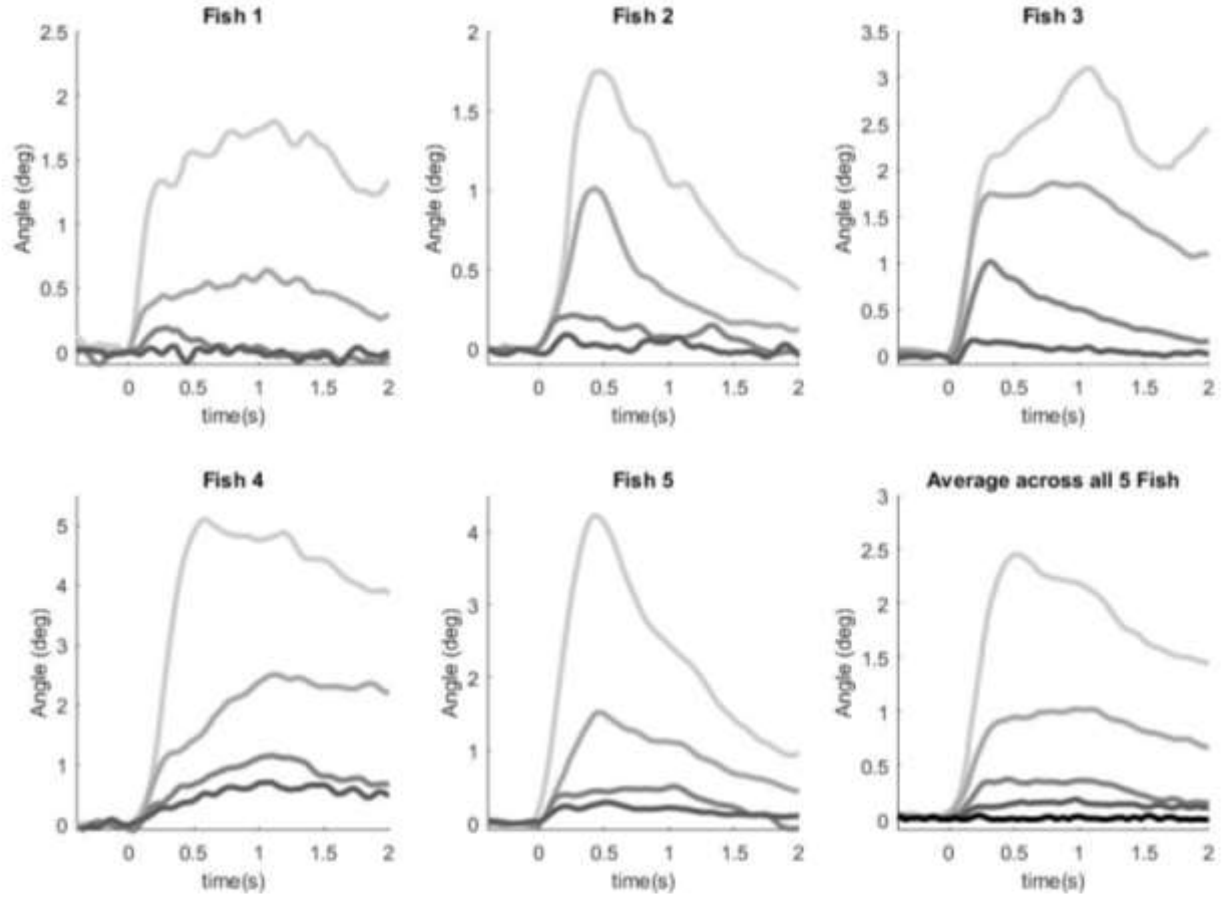

Supplementary Figure 4: Tail responses of all fish to an OT on the lateral edge of one otolith. Tail response curves for each of the 5 fish and the average across all 5 fish**.** Data are represented as in Fig 3c. Powers are 600, 400, 200, and 100 mW (light to dark curves).

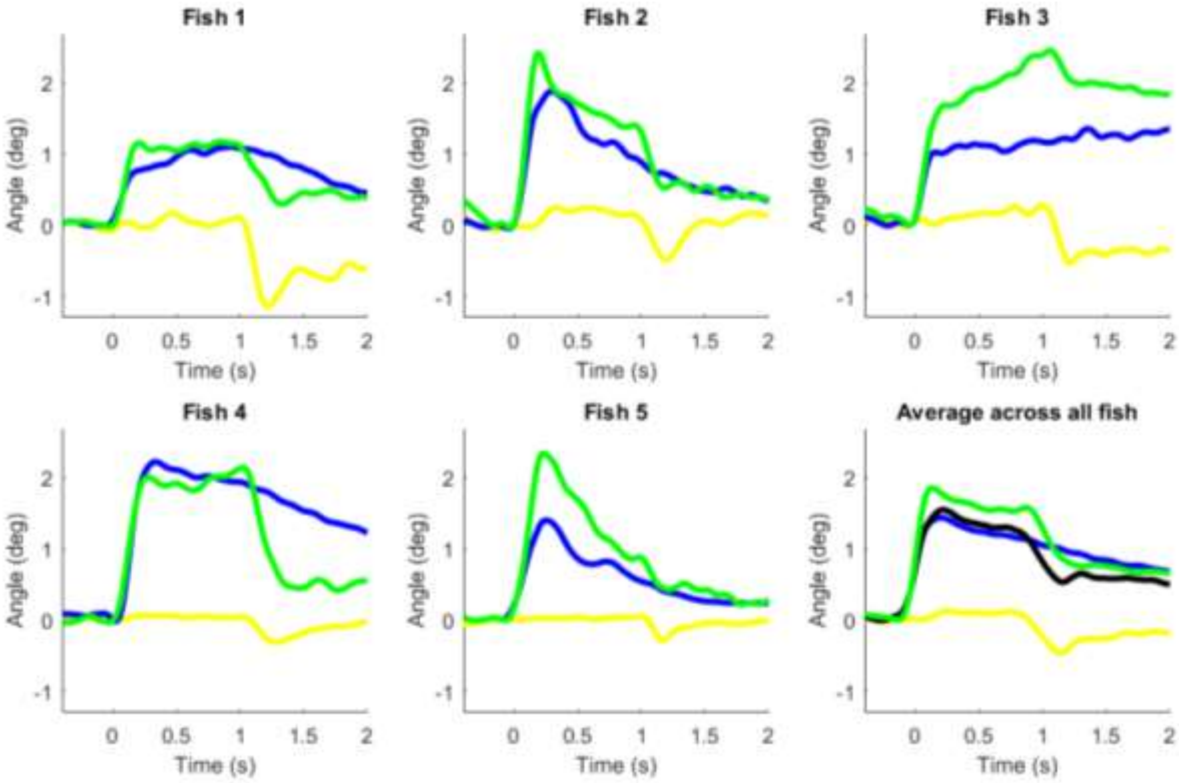

Supplementary Figure 5: Tail responses of all fish to dual OT. Tail response curves for each of the 5 fish and the average across all 5 fish**.** Data are represented as in Fig 3f. Blue curve, ROlat; yellow curve, LOmed; green curve, ROlat and LOmed; black curve, sum of ROlat and LOmed. These data produce the values shown in Fig 3g.

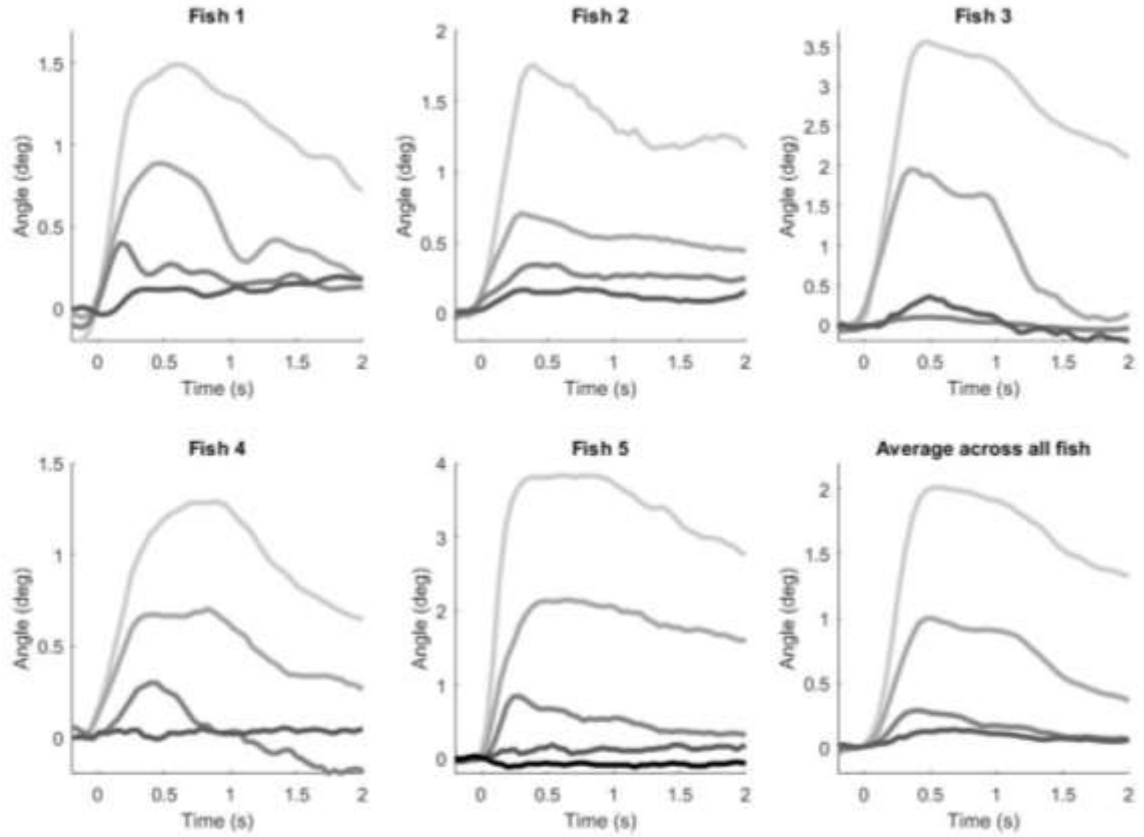

Supplementary Figure 6: Eye responses of all fish to an OT on the lateral edge of one otolith. Eye response curves for each of the 5 fish and the average across all 5 fish**.** Data are represented as in Fig 3j. Powers are 600, 400, 200, and 100 mW (light to dark curves).

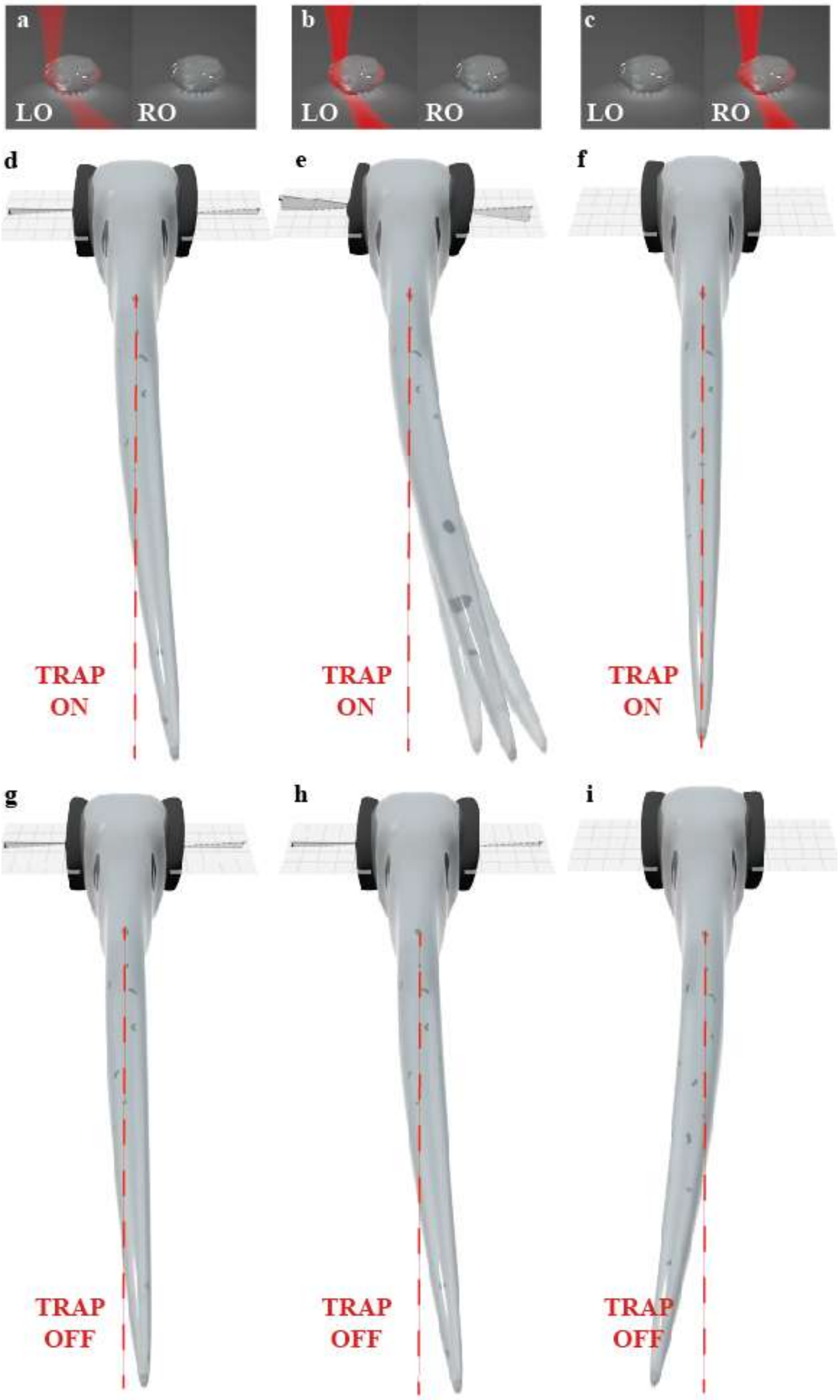

Supplementary Figure 7: Summary of behavioural responses to OT of the otoliths. Here, we present a review and summary of the behavioural responses elicited by weak lateral, strong lateral, and strong medial otolith traps, as reported in Figures 3 and 4, and in Extended Data Figures 2 and 3. **a,** A weak OT on the exterior of one otolith causes a mild contralateral bend of the tail and a slight roll of the eyes **(d)**, which passively return to baseline when the OT is terminated **(g)**. A stronger trap of the same type **(b)** drives a more dramatic bend and a forward swim in the tail, and a more dramatic eye roll **(e)**, and again, these passively return to baseline from these positions **(h)**. A trap to the medial edge of the opposite otolith **(c)** produces no behavioural response **(f)**, while the offset of this OT produces a contralateral tail bend **(i)**.

Supplementary References

1 Westerfield, M. *The Zebrafish Book*. 5th edn, (University of Oregon Press, 2007).
2 Lister, J. A., Robertson, C. P., Lepage, T., Johnson, S. L. & Raible, D. W. nacre encodes a zebrafish microphthalmia-related protein that regulates neural-crest-derived pigment cell fate. *Development* **126**, 3757-3767 (1999).
3 Favre-Bulle, I. A. *et al.* Scattering of Sculpted Light in Intact Brain Tissue, with implications for Optogenetics. *Scientific Reports* **5**, 11501 (2015).
4 Allen, L., Beijersbergen, M. W., Spreeuw, R. J. C. & Woerdman, J. P. Orbital angular momentum of light and the transformation of Laguerre-Gaussian laser modes. *Physical Review A* **45**, 8185-8189 (1992).
5 Böhmer, M. & Enderlein, J. Orientation imaging of single molecules by wide-field epifluorescence microscopy. *J. Opt. Soc. Am. B* **20**, 554-559, doi:10.1364/JOSAB.20.000554 (2003).
